# 1474. Getting to Zero: Our Journey to Decrease CABG Deep Sternal Wound Infections

**DOI:** 10.1093/ofid/ofad500.1310

**Published:** 2023-11-27

**Authors:** Paul M Gentile, Scott Roberts, Erin Wilson, Richard A Martinello

**Affiliations:** Yale New Haven Hospital, New Haven, Connecticut; Yale New Haven Health, New Haven, Connecticut; Yale New Haven Hospital, New Haven, Connecticut; Yale School of Medicine, New Haven, Connecticut

## Abstract

**Background:**

Deep sternal wound infections (DSWI) are a serious complication of cardiac thoracic surgeries and are associated with a significantly higher mortality risk. Our center experienced increased surgical site infection (SSI) rates prompting an epidemiologic evaluation.

**Methods:**

A retrospective cohort review of 10 cardiac surgery patients with DWSI from 2020-2021 were reviewed. An SSI bundle to decrease DSWI was implemented using a standardized pos-operative wound dressing (prior to this, dressing selection was per the cardiac thoracic surgeon's discretion), glucose optimization in collaboration with our endocrinology team, and chlorhexidine bathing practices.

**Results:**

Starting in September 2020 Yale New Haven Hospital observed a statistically significant increase in DSWI (0.51), which was a 285% increase in DSWI. Of the 10 out of 15 patients with DSWI who underwent review, the majority were inpatient prior to surgery with a mean length of stay of 5 days before undergoing surgery. Following selective implementation of the SSI bundle to urgent cases who were inpatient at the time of surgery, the DSWI rate decreased from 1.97 in 2021 to 1.13 in 2022, which reflected a 43% clinical decrease with 0 DSWI reported.

**Conclusion:**

Implementation of an inpatient pre-operative, SSI bundle, including standardization of post-operative sternal wound dressings, resulted in a significant decrease in DSWI that has persisted post-intervention.

**Disclosures:**

**All Authors**: No reported disclosures

